# Activation of mesenchymal stem cells promotes new bone formation within dentigerous cyst

**DOI:** 10.1186/s13287-020-01999-8

**Published:** 2020-11-10

**Authors:** Yejia Yu, Mengyu Li, Yuqiong Zhou, Yueqi Shi, Wenjie Zhang, Geehun Son, Jing Ge, Jun Zhao, Zhiyuan Zhang, Dongxia Ye, Chi Yang, Shaoyi Wang

**Affiliations:** 1 Department of Oral Surgery, Shanghai Key Laboratory of Stomatology & Shanghai Research Institute of Stomatology, National Clinical Research Centre for Oral Diseases, Ninth People’s Hospital, Shanghai Jiao Tong University School of Medicine, Shanghai, China; 2 Department of Prosthodontics, Shanghai Engineering Research Centre of Advanced Dental Technology and Materials, Shanghai Key Laboratory of Stomatology & Shanghai Research Institute of Stomatology, National Clinical Research Centre for Oral Diseases, Ninth People’s Hospital, Shanghai Jiao Tong University School of Medicine, Shanghai, China; 3 Department of Orthodontics, Shanghai Key Laboratory of Stomatology & Shanghai Research Institute of Stomatology, National Clinical Research Centre for Oral Diseases, Ninth People’s Hospital, Shanghai Jiao Tong University School of Medicine, Shanghai, China; 4 Department of Oral-maxillofacial Head and Neck Oncology, Shanghai Key Laboratory of Stomatology & Shanghai Research Institute of Stomatology, National Clinical Research Centre for Oral Diseases, Ninth People’s Hospital, Shanghai Jiao Tong University School of Medicine, Shanghai, China; 5 Shanghai Key Laboratory of Stomatology & Shanghai Research Institute of Stomatology, National Clinical Research Centre for Oral Diseases, Ninth People’s Hospital, Shanghai Jiao Tong University School of Medicine, Shanghai, China

**Keywords:** Dentigerous cyst, Mesenchymal stem cells, Osteogenic differentiation, Bone regeneration, Cell proliferation, Cell culture

## Abstract

**Background:**

Dentigerous cyst (DC) is a bone destructive disease and remains a challenge for clinicians. Marsupialization enables the bone to regenerate with capsule maintaining, making it a preferred therapeutic means for DC adjacent to vital anatomical structures. Given that capsules of DC are derived from odontogenic epithelium remnants at the embryonic stage, we investigated whether there were mesenchymal stem cells (MSCs) located in DC capsules and the role that they played in the bone regeneration after marsupialization.

**Methods:**

Samples obtained before and after marsupialization were used for histological detection and cell culture. The stemness of cells isolated from fresh tissues was analyzed by morphology, surface marker, and multi-differentiation assays. Comparison of proliferation ability between MSCs isolated from DC capsules before (Bm-DCSCs) and after (Am-DCSCs) marsupialization was evaluated by Cell Counting Kit-8 (CCK-8), fibroblast colony-forming units (CFU-F), and 5′-ethynyl-2′-deoxyuridine (EdU) assay. Their osteogenic capacity in vitro was detected by alkaline phosphatase (ALP) and Alizarin Red staining (ARS), combined with real-time polymerase chain reaction (RT-PCR) and immunofluorescence (IF) staining. Subcutaneous ectopic osteogenesis as well as cranial bone defect model in nude mice was performed to detect their bone regeneration and bone defect repairability.

**Results:**

Bone tissue and strong ALP activity were detected in the capsule of DC after marsupialization. Two types of MSCs were isolated from fibrous capsules of DC both before (Bm-DCSCs) and after (Am-DCSCs) marsupialization. These fibroblast-like, colony-forming cells expressed MSC markers (CD44+, CD90+, CD31−, CD34−, CD45−), and they could differentiate into osteoblast-, adipocyte-, and chondrocyte-like cells under induction. Notably, Am-DCSCs performed better in cell proliferation and self-renewal. Moreover, Am-DCSCs showed a greater osteogenic capacity both in vitro and in vivo compared with Bm-DCSCs.

**Conclusions:**

There are MSCs residing in capsules of DC, and the cell viability as well as the osteogenic capacity of them is largely enhanced after marsupialization. Our findings suggested that MSCs might play a crucial role in the healing process of DC after marsupialization, thus providing new insight into the treatment for DC by promoting the osteogenic differentiation of MSCs inside capsules.

## Background

As one of the developmental cysts, dentigerous cyst (DC) accounts for approximately 20% of odontogenic cysts, ranking second in major common cysts in the oral and maxillofacial region [1]. It is often diagnosed clinically by a radiographic characteristic that the capsule attaches to the cemento-enamel junction (CEJ) with the crown of an unerupted tooth enclosed. The enlarged cyst usually results in dentition disturbance, dentofacial deformity, and even pathological fracture [2, 3]. The standard treatment is surgical enucleation or curettage of the capsule combined with the involved tooth, while marsupialization is preferred in some pediatric cases and large cystic lesions (radiologic diameter > 3 cm), especially when they are proximal to vital structures [4–7]. However, it takes quite a long time for DC to reach recovery under marsupialization. To shorten the course of treatment, suction drainage has been applied to the clinic, but problems are still tough about the inconvenience and infection brought by suction drainage devices [8].

The healing of DC is a process of new bone formation. It is reported that MSCs are of great importance during the bone formation process because of their distinct biological capability in bone regenerative medicine [9]. Dental tissues have been considered as promising sources for stem cells since MSCs such as DPSCs, PDLSCs, and SCAPs were isolated one after another [10–12]. Odontogenesis is based on the organized reciprocal interaction of the odontogenic epithelial and neural crest-derived tissues [13]. Tooth initiation starts at the 6th embryonic week, and the primary epithelial band differentiates into the vestibular and dental lamina 1 week later. The latter undergoes 3 stages (bud stage, cap stage, and bell stage) and gives rise to the enamel organs to form tooth enamel [14]. DC develops as a result of fluid accumulation between the crown of the unerupted tooth and reduced enamel epithelium, which is known as the remnants of enamel organs. That is, capsules of DC originate from the remnants of enamel organs at the embryonic stage. A series of studies have shown that rodent incisors grow continuously throughout life owing to the epithelial progenitor cells in the cervical loop, an area where the inner and the outer enamel epithelium meet at the rim of the enamel organ at the bell stage [15–17]. Besides, stem cell markers such as Oct-4, CD44, and K15 have been demonstrated in odontogenic lesions as well [18, 19]. In addition, odontogenic epithelium, such as remnants of the dental lamina, the epithelial cell rests of Malassez, and reduced enamel epithelium, has been thought to be hidden sources in regenerative medicine because of the existence of stem cells in them [20]. What is more, Marrelli et al. have isolated MSC-like cells from human periapical cysts [21]. Evidences above preliminary suggest the possibility that there are stem cells in the capsule of DC.

The intra-cystic pressure is released after marsupialization, and changes occur in the cystic lining as well. The thickened capsule has been demonstrated to turn into a less aggressive form [22]. What is more, osteogenesis-related proteins, such as ALP, BMP2, BMP4, and OPN, have shown upregulations in capsules proximal to the bone after marsupialization [23]. Based on the researches above, we inferred that the post-marsupialization cyst capsule may be a microenvironment which is conducive to the osteogenic differentiation of stem cells.

To our knowledge, it has not yet been reported about the presence of stem cells in DC capsules and their role in the healing process after marsupialization up to now. In this study, we isolated two types of cells from the connective tissue of DC capsules before (Bm-DCSCs) and after marsupialization (Am-DCSCs), respectively. After that, we compared their stemness by surface markers, multi-differentiation potential assays, proliferation, and self-renewal capacity assays, ectopic bone regeneration assay, and bone defect repairability in the nude cranial defect model.

## Materials and methods

### Sample collection

**Fig. 1:**
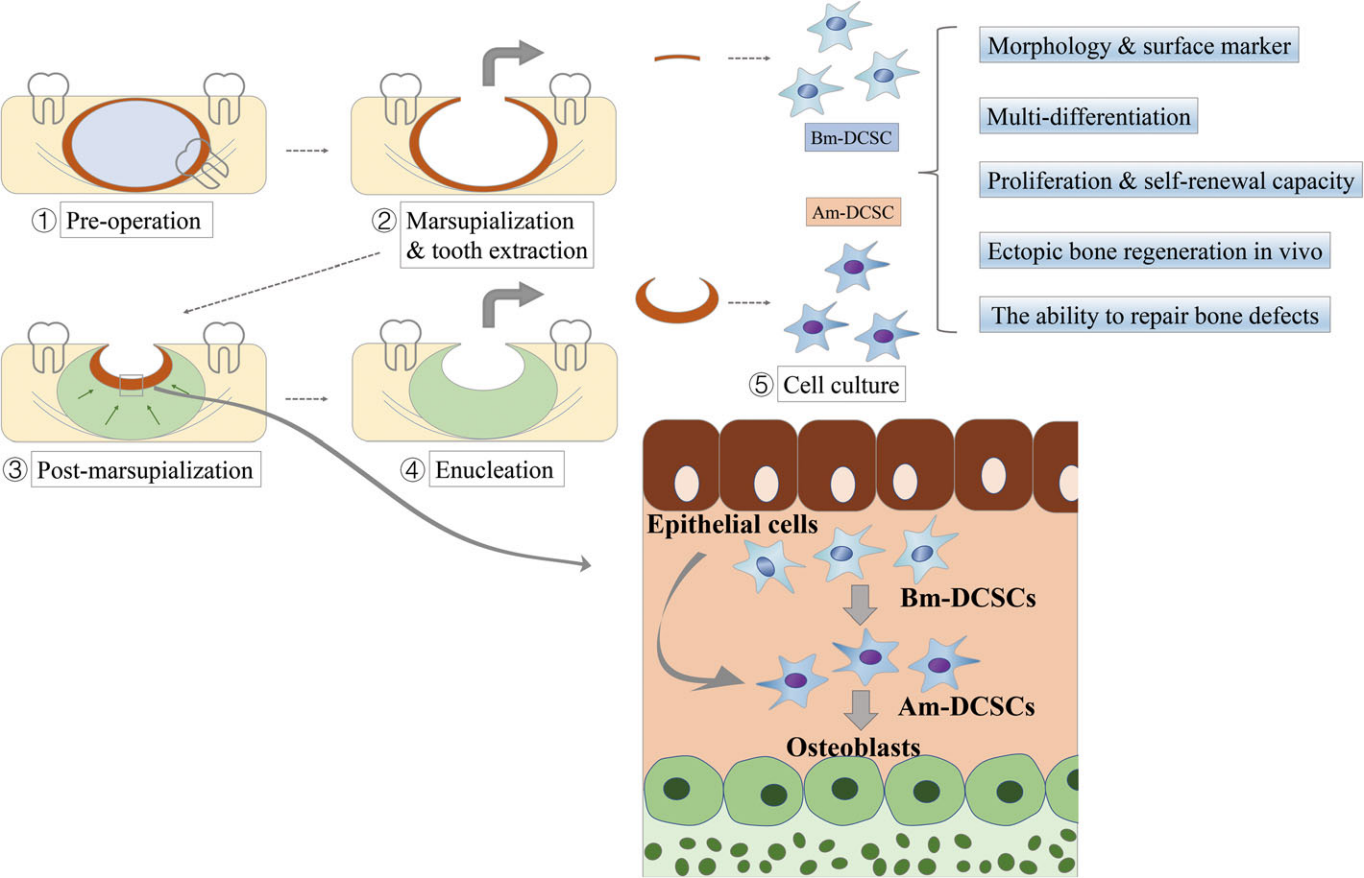
Schematic illustration of sample collection and cell culture. The DC adjacent to the inferior alveolar nerve underwent marsupialization and tooth extraction. A few months later, the formation of new bone decreased the size of DC (green area). The remaining capsule of DC became thickened and was removed for medical demand. The capsule tissue both before and after marsupialization was collected for histology analysis and cell culture

Five patients who underwent marsupialization combined enucleation later were included in this study (Table 1), and the tissue sampling process was illustrated in Fig. 1. Samples harvested before and after marsupialization were stored in 4% paraformaldehyde for 6–8 h and high-glucose Dulbecco’s modified Eagle’s medium (DMEM; HyClone) supplemented with 100 U/ml penicillin and 100 mg/ml streptomycin (HyClone) at 4 °C up to 2 h, respectively. Patients engaged in this trial aged between 18 and 25 years, who were diagnosed as DC both clinically and histologically. This work was approved by the Ethics Committee of Shanghai Ninth People’s Hospital, and all participants gave their informed consent.

**Table 1:** Duration of marsupialization

No	Age	Gender	Duration of marsupialization
1	18	F	8 M
2	20	M	7.5 M
3	25	M	10 M
4	22	F	9 M
5	19	F	11 M

### Histological analysis

Capsules fixed in 4% paraformaldehyde were embedded in paraffin and sliced for histological evaluation. Paraffin sections were stained with hematoxylin and eosin (H&E) as well as Safranin O/Fast Green. For immunofluorescent staining, after deparaffinization, rehydration, antigen retrieval, permeabilization, and blocking non-specific binding, sections were incubated in primary antibodies against STRO-1 (Novus Biologicals; 1:100), ALP (Servicebio; 1:3000), COL1A1 (Servicebio; 1:800) at 4 °C overnight and secondary antibodies (Servicebio; 1:500) for 1 h at room temperature. DAPI (Abcam) at 1:500 was used as a nuclear counterstain. Results were detected by a fluorescence microscope (Olympus).

### Cell culture

Fresh samples were rinsed with phosphate-buffered saline (PBS) for three times and then gently minced into small pieces. The fragments were transferred to 12-well chambers (Coster) and 100-mm dishes (Corning) with complete medium—DMEM containing 10% fetal bovine serum (FBS; Every Green), 100 U/ml penicillin and 100 mg/ml streptomycin (HyClone). The glass cover slips were placed over the fragments to prevent floating. Cells were cultured at 37 °C with 5% CO_2_, and the culture medium was changed twice a week. Bm-DCSCs and Am-DCSCs would be harvested and amplified when reaching 80–90% confluence, and cells at passages 2–4 were used in this work. The primary passage of Am-DCSCs on the glass cover slips in 12-well chambers was used for immunofluorescence staining.

### Morphology and surface marker

#### Immunofluorescence

The primary passage of cells in 12-well chambers was fixed in 4% paraformaldehyde for 30 min at room temperature. After that, Am-DCSCs were permeabilized in 0.3% Triton X-100 for 5 min and blocked in 5% BSA for 1 h. Then, Am-DCSCs were incubated with antibodies against STRO-1 (Novus Biologicals) at 1:150 dilution at 4 °C overnight. Alexa Fluor-648-conjugated anti-IgM (Yeasen) was used as a secondary antibody at 1:200 dilution, and DAPI (Abcam; 1:500) was used for nuclear counterstain. Bm-DCSCs and Am-DCSCs at P_3_ were stained by Alexa Fluor 555 Phalloidin (Abcam; 1:200) and DAPI (Abcam; 1:500) as well for actin staining. Slides were examined with a confocal laser scanning microscope (CLSM; Leica).

#### Flow cytometry analysis

Both Bm-DCSCs and Am-DCSCs were detached with 0.25% trypsin-EDTA (Gibco) for 20–30 s and centrifuged at 1000 rpm for 5 min. After removing the supernatant, cells were washed twice by PBS, and then, they were collected and resuspended in PBS. Cell surface markers, such as CD90-FITC, CD44-FITC, CD45-FITC, CD34-FITC, and CD31-FITC, were used to label the cells on ice for 30 min in the dark. Cell suspensions without the antibodies served as controls. Cells were washed twice and resuspended in 200 μL PBS before analysis. All antibodies were purchased from BD Biosciences. Flow cytometry was performed with a flow cytometer (Beckman Coulter).

#### Multi-differentiation assays

Bm-DCSCs and Am-DCSCs were seeded into 12-well plates and cultured in the complete medium until 100% confluence. Osteogenic differentiation medium, containing 2 mmol/L β-glycerophosphate (Sigma-Aldrich), 100 mmol/L L-ascorbic acid phosphate (Sigma-Aldrich), and 10 nmol/L dexamethasone, was used for osteogenic induction. Adipogenic and chondrogenic induction were stimulated by commercial kits (Cyagen) according to the manufacturer’s instructions. Osteogenesis effects were detected by alkaline phosphatase (ALP; Beyotime) after 1, 3, and 7 days and Alizarin Red staining (ARS; Sigma-Aldrich) after 7, 14, and 21 days, and the quantitative assays were performed at the same time. Besides, Oil Red O and Alcian blue were used for adipogenic and chondrogenic detection after 3–4 weeks’ induction.

#### Real-time PCR

The gene expression in Bm-DCSCs and Am-DCSCs after osteogenic differentiation was detected by real-time PCR. Total RNA was extracted with RNAiso Plus (TaKaRa), and complementary DNA (cDNA) was synthesized using the PrimeScriptT^M^ RT reagent kit (TaKaRa). The housekeeping gene GAPDH was used for normalization. Primers were synthesized commercially (Shengong). The sequences of the primers were as follows: GAPDH-CGACAGTCAGCCGCATCTT and CCAATACGACCAAATCCGTTG, RUNX2-TCTTAGAACAAATTCTGCCCTTT, and TGCTTTGGTCTTGAAATCACA, OCN-GGCAGCGAGGTAGTGAAGA, and TCAGCCAACTCGTCACAGTC.

#### Immunofluorescence

The osteogenic protein expression of Bm-DCSCs and Am-DCSCs was detected by immunofluorescence. After 7 days’ osteogenic induction, cells were permeabilized in 0.3% Triton X-100 for 5 min and blocked in 5% BSA for 1 h. Then, they were incubated with antibodies against osteocalcin (OCN; Abcam) and RUNX2 (CST) at 1:100 dilution at 4 °C overnight. After that, cells were incubated with secondary antibodies (1:500; Invitrogen) for 30 min and subsequently incubated with DAPI (1:500; Abcam) for 5 min at room temperature. The undifferentiated cells were served as controls. Results were detected by a fluorescence microscope (Olympus).

### Proliferation and self-renewal capacity

#### Cell proliferation

Bm-DCSCs and Am-DCSCs were seeded in 96-well plates (Costar) at a density of 1000 cells per well. The cell number was assessed on days 1, 3, 5, 7, 9, and 11 with the Cell Counting Kit-8 (Dojindo Laboratories). The optical density was measured at a wavelength of 450 nm using the Spark™ 10 M Multimode Microplate Reader (TECAN).

#### Colony-forming unit

Cells were seeded in 6-well plates at a density of 100 cells per well. After 10 days’ culture, they were fixed with 4% paraformaldehyde and stained with 0.1% crystal violet (Beyotime) for 5 min, and aggregates of 50 or more cells were scored as colonies.

#### EdU Assay

EdU Assay was detected by BeyoClick™ EdU Cell Proliferation Kit with Alexa Fluor 555 (Beyotime). Firstly, Bm-DCSCs and Am-DCSCs were seeded in 12-well plates. 24 h later, cells were labeled by EdU for 2 h, and then, they were fixed and permeabilized. Solution for EdU detection was prepared according to the manual, and cells were analyzed by a fluorescence microscope. Besides, the number of EdU-positive cells was detected by flow cytometry for quantitative analysis.

### Ectopic bone regeneration in vivo

#### Scanning electronic microscopy

Cells were seeded onto the surface of β-tricalcium phosphate (β-TCP; Shanghai Bio-Lu Biomaterials Co., Ltd.) at a density of 1.0 × 10^6^/ml. After incubation for 4 h and 1 day, the scaffolds were fixed in 2.5% glutaraldehyde overnight at 4 °C. Then, they were dehydrated through an ethanol gradient (30%, 50%, 70%, 90%, and 100% for twice) for 10 min in each concentration. After that, they were transferred to the mixture of alcohol and iso-amyl acetate (V/V = 1:1) for 30 min and pure iso-amyl acetate for 1 h. Followed by a critical point dryer with liquid CO_2_, samples were coated with gold sputter and images were collected by a scanning electron microscope.

#### In vivo ectopic transplantation model

β-tricalcium phosphate mixed with 5.0 × 10^6^ of Bm-DCSCs or Am-DCSCs were transplanted into aseptically created subcutaneous pockets in 6-week-old immunocompromised mice under anesthesia via 2% sodium pentobarbital. β-TCP with PBS were seeded on the other side of the dorsum in the same mice in order to serve as a control group. Transplants were harvested after 8 weeks and assessed by histology.

### The ability to repair bone defect in situ

#### Cranial bone defect model in immunocompromised mice

β-tricalcium phosphate was mixed with 5.0 × 10^6^ of Bm-DCSCs or Am-DCSCs. Immunocompromised mice were anesthetized via 2% sodium pentobarbital. Then, a sagittal incision was created in the middle of the scalp. After exposing the calvarium, the periosteum was carefully deflected with ophthalmic forceps. A 5-mm-size defect was made on the calvarium using a trephine with constant sterile saline cooling. Finally, the scaffold was implanted within the defect, and the periosteum as well as the scalp was repositioned and sutured. β-TCP with PBS were served as the control group. Transplants were harvested after 12 weeks and assessed by histology.

#### Sequential fluorescent labeling

To label the rate of new bone formation, sequential fluorescent labeling was carried out on 3 athymic mice from each group. They were intraperitoneally injected with 25 mg/kg hydrochloride tetracycline (TE, Sigma), 20 mg/kg calcein (CA, Sigma), and 30 mg/kg Alizarin Red S (AL, Sigma) at 3, 6, and 9 weeks after surgery, respectively. The calvarias were harvested at 12 weeks and used for non-decalcified tissue histomorphometric measurements. Mineral apposition rate (μm/day) was measured and evaluated [24–26].

### Statistical analysis

Results were presented as the mean ± standard deviation. The statistical analysis was performed using the GraphPad Prism statistical software package (Version 7.0). One-way ANOVA was performed, followed by Dunnett’s test for multiple comparisons (*p < 0.05; ***p < 0.001; ****p < 0.0001).

## Results

### STRO-1-positive cells resided in the DC capsule after marsupialization (Am-DCC) which was a microenvironment conducive to osteogenesis

**Fig. 2:**
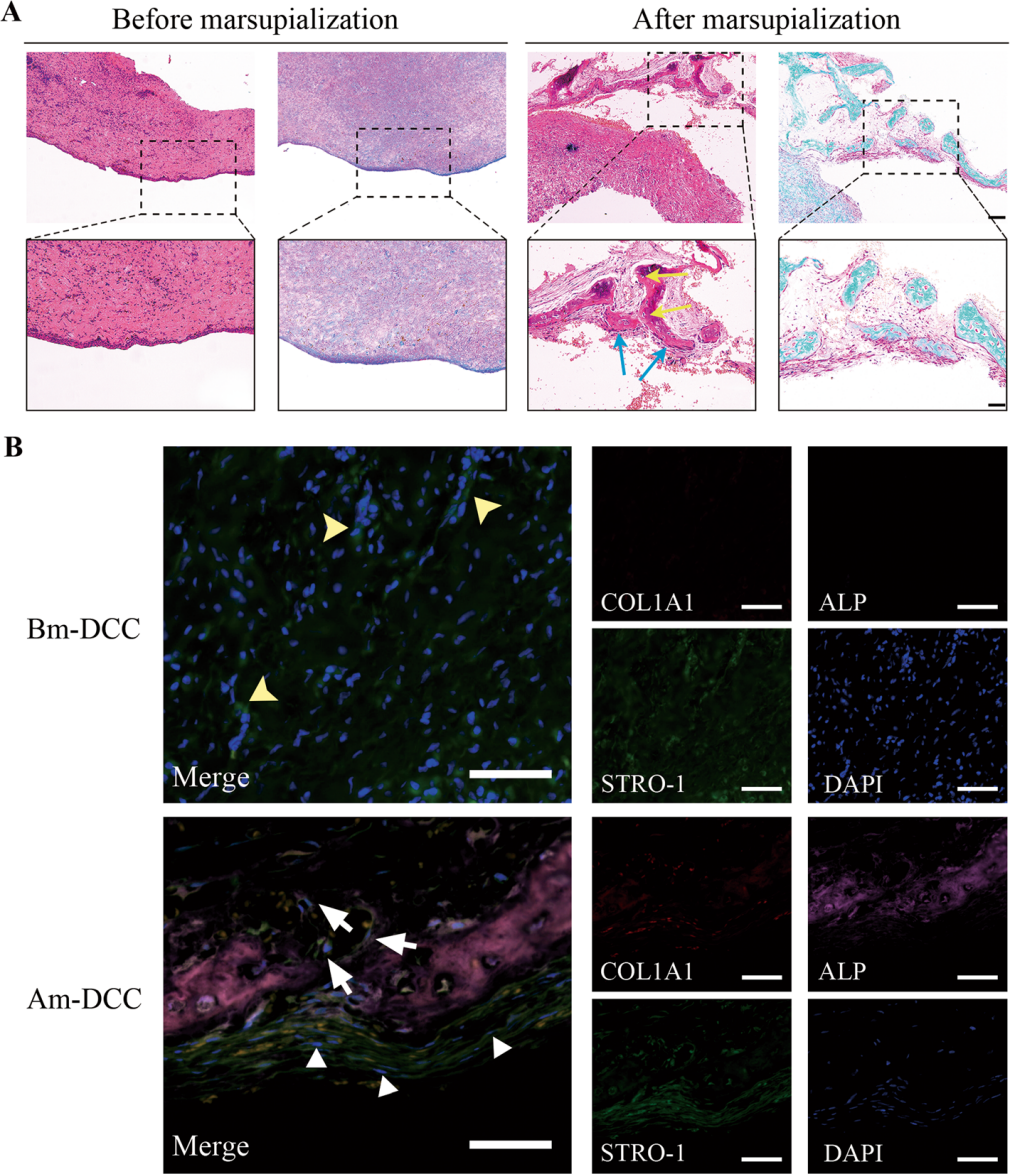
Histologic analysis of DC capsules before and after marsupialization. **a** The capsules were detected by hematoxylin-eosin staining (H&E) and Safranin O/Fast Green. Compared with DC capsules before marsupialization (Bm-DCC), DC capsules after marsupialization (Am-DCC) lost the epithelium lining and the fiber became loose. Clumps (which were demonstrated as the bone tissues) were observed in Am-DCC, in which osteocytes (yellow arrows) and osteoblasts (blue arrows) could be seen as well. Boxed areas are shown at higher magnification. Scale bars: 100 μm and 50 μm. **b** Representative immunofluorescence images of Bm-DCC and Am-DCC. STRO-1-positive cells (yellow arrows) were detected in the fibrous connective tissue of Bm-DCC. STRO-1-positive cells (white triangles) and ALP-positive cells (white arrows) were detected in the fibrous connective tissue of Am-DCC. DAPI (blue) was counterstained to indicate the nucleus. Scale bars: 50 μm

From the section stained by hematoxylin and eosin (H&E), the cylindrical epithelium of the capsule after marsupialization was missing owing to the repeated inflammation stimuli compared with the tissue before the surgery. However, clumps were observed in the layer of fibrous connective tissue of Am-DCC adjacent to the bone, which were demonstrated as the bone tissue by Safranin O/Fast Green stain later (Fig. 2a). In order to further investigate the origin of the bone tissue, immunofluorescence (IF) staining of COL1A1, ALP, and STRO-1 was performed on Am-DCC. As shown in Fig. 2b, the bone tissue was slightly positive for COL1A1, while they were strongly stained by ALP, demonstrating that the bone tissue was at an early stage. Meanwhile, we found that the fibro-cellular connective tissue of Am-DCC was labeled by ALP, which means the microenvironment of Am-DCC was conducive to osteogenesis. Moreover, fibrous connective tissue cells around the bone tissue were labeled by STRO-1, an early MSC marker, indicating the source of bone tissue. We then detected the DC capsule before marsupialization (Bm-DCC) and found STRO-1-positive cells as well, while ALP-positive and COL1A1-positive cells were not observed.

### Am-DCSCs and Bm-DCSCs showed typical MSC morphological characteristics and immunophenotype

**Fig. 3:**
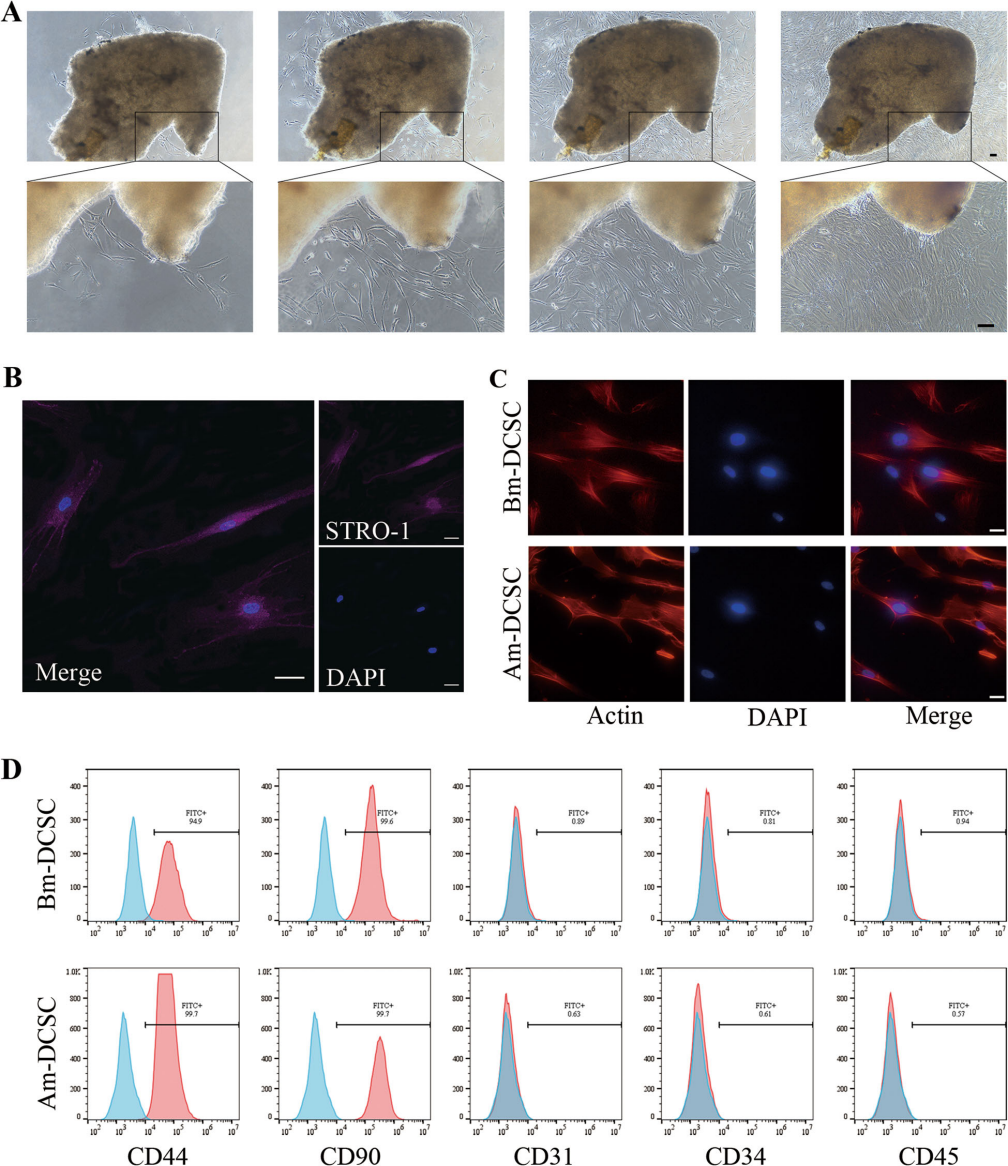
Morphological characteristics and immunophenotype of Bm-DCSCs and Am-DCSCs. **a** Representative images of cells isolated from DC capsules at different magnifications under inverted phase-contrast microscope. Scale bars: 100 μm. **b** Immunofluorescence detection of STRO-1 (violet) on Am-DCSCs. Scale bar: 50 μm. **c** Bm-DCSCs and Am-DCSCs showed spindle-shaped morphology under a fluorescence microscope labeled by actin (red). Scale bar: 50 μm. **d** Flow cytometry data of MSC markers on Bm-DCSCs and Am-DCSCs

Am-DCSCs showed fibroblast-like appearance (Fig. 3a, c). According to flow cytometry (FCM) results (Fig. 3d), Am-DCSCs showed positive expression of the cell surface antigens CD44 (99.7%) and CD90 (99.7%), while a lack of expression of hematopoietic antigens including CD31 (0.63%), CD34 (0.61%), and CD45 (0.57%). It highlighted that cells isolated from Am-DCC showed both MSC appearance and surface markers. Besides, Am-DCSCs expressed STRO-1, which was also detected in the connective tissue adjacent to the bone tissue in Am-DCC (Fig. 3b). Results above indicated that there were MSC-like cells in Am-DCC and they could be isolated from it with characteristics maintaining. Interestingly, we isolated fibroblast-like cells in the DC capsule before marsupialization (Bm-DCC) as well, and they showed similar immunophenotype as Am-DCSCs (Fig. 3c, d). What emerged from the results here was that cells with MSC feature could be isolated from DC capsules both before and after marsupialization.

### Am-DCSCs showed greater osteogenic differentiation capacity than that of Bm-DCSCs in vitro

**Fig. 4:**
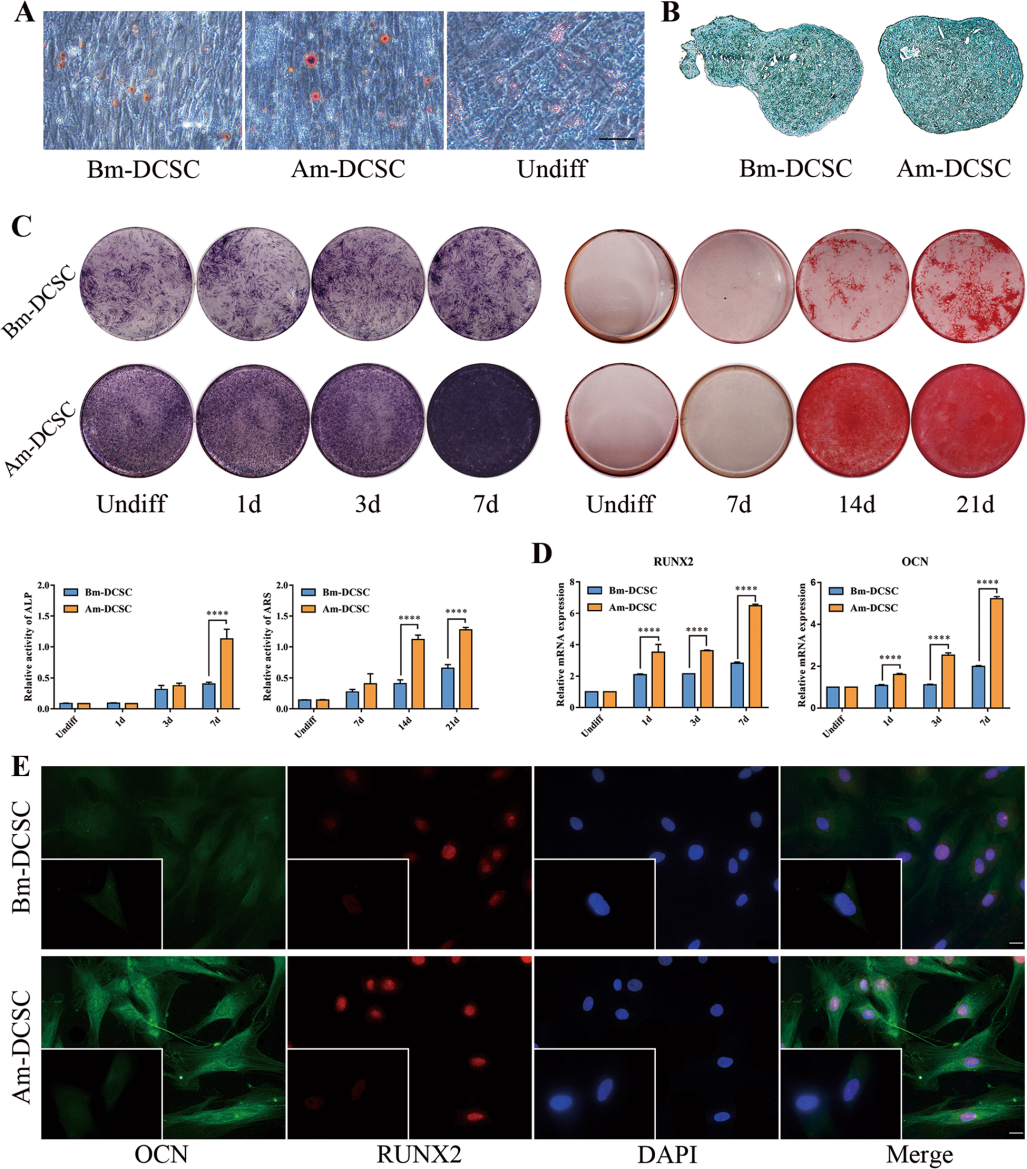
Bm-DCSCs and Am-DCSCs exhibited a multi-differentiation potential. **a** Oil Red O staining. **b** Alcian blue staining. **c** Alkaline phosphatase staining (ALP) and Alizarin Red staining (ARS) at different osteogenic induction time. Relative activity of ALP and ARS were detected at the same time. ****p < 0.0001. **d** Real-time polymerase chain reaction (RT-PCR) for messenger RNA (mRNA) expression levels of runt-related transcription factor 2 (Runx2) and osteocalcin (OCN). ****p < 0.0001. **e** Immunofluorescence staining for OCN and RUNX2 on Bm-DCSCs and Bm-DCSCs after 7 days’ osteogenic differentiation. Scale bars: 20 μm. The undifferentiated cells were shown at the lower left corner in each image with a magnification of × 1000

The trilineage differentiation assay was performed to determine whether Am-DCSCs or Bm-DCSCs had the ability to differentiate into multiple tissues when cultured under specific conditions. After 3 weeks’ adipogenic induction, Oil Red O-positive lipid-laden fat cells could be detected in both Bm-DCSCs and Am-DCSCs by inverted phase-contrast microscope (Fig. 4a), and Alcian blue staining demonstrated the existence of cartilage tissues differentiated from Bm-DCSCs as well as Am-DCSCs (Fig. 4b). However, we observed that Am-DCSCs displayed stronger ALP activity on day 7 and better calcium deposition ability on days 14 and 21 compared with Bm-DCSCs after osteogenic induction (Fig. 4c). Meanwhile, the expression of osteogenesis-related genes, such as RUNX2 and OCN, was significantly increased in Am-DCSCs on days 3 and 7 after induction, and this development correlated with the protein expression of them detected by IF staining (Fig. 4d, e). All the results above indicated that both Bm-DCSCs and Am-DCSCs had multi-differentiation potential, while the latter showed greater osteogenic differentiation capacity in vitro.

### Am-DCSCs showed better proliferation and self-renewal capacity than that of Bm-DCSCs

**Fig. 5:**
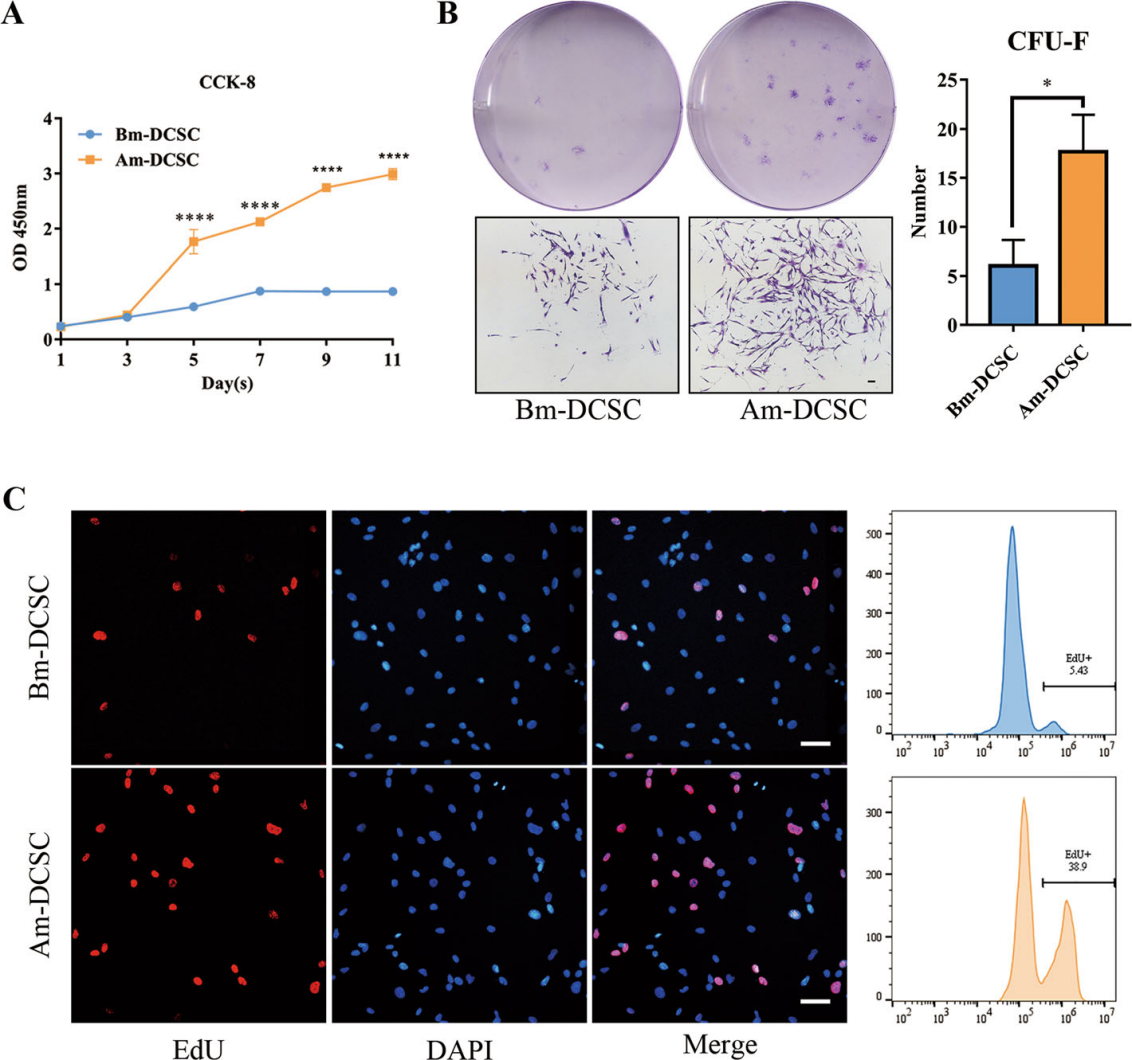
Am-DCSCs showed better proliferation and self-renewal capacity than that of Bm-DCSCs in vitro. **a** Cell viability of Bm-DCSCs and Am-DCSCs was detected by CCK-8 assay. ****p < 0.0001. **b** Colony-forming unit-fibroblast (CFU-F) assay was stained by crystal violet. Typical clusters were observed in both Am-DCSCs and Bm-DCSCs. Scale bar: 100 μm. Aggregates of 50 or more cells were scored as colonies. *p < 0.05. **c** Proliferative cell nucleus was labeled by EdU. Scale bars: 50 μm. Quantitative analysis of EdU-positive cells was detected by FCM

To assess the ability of rapid proliferation and self-renewal of Bm-DCSCs and Am-DCSCs, assays such as CCK-8, CFU-F, and EdU were conducted. According to CCK-8 (Fig. 5a), Am-DCSCs showed greater cell proliferation ability than that of Bm-DCSCs. Bm-DCSCs were in a slow proliferation state between days 1 and 7 and reached a plateau after day 7. However, Am-DCSCs were in a latent period between days 1 and 3, entering a logarithmic growth phase between days 3 and 5, and they showed a high proliferation rate during the later stage. Besides, although both Bm-DCSCs and Am-DCSCs formed typical clusters after 10 days’ culture at a low inoculation density, the number of cell colonies in the Am-DCSC group (17.67 ± 2.186) was significantly higher than that in the Bm-DCSC group (6 ± 1.582) (Fig. 5b). We could also see proliferative cell nucleus labeled by EdU in Bm-DCSCs and Am-DCSCs shown by a fluorescence microscope, while the proliferation rate in Am-DCSCs was higher than that in Bm-DCSCs detected by FCM (Fig. 5c). Collectively, it could be concluded that Am-DCSCs showed better proliferation and self-renewal capacity compared with Bm-DCSCs.

### Am-DCSCs showed greater ectopic bone regeneration capacity than that of Bm-DCSCs in vivo

**Fig. 6:**
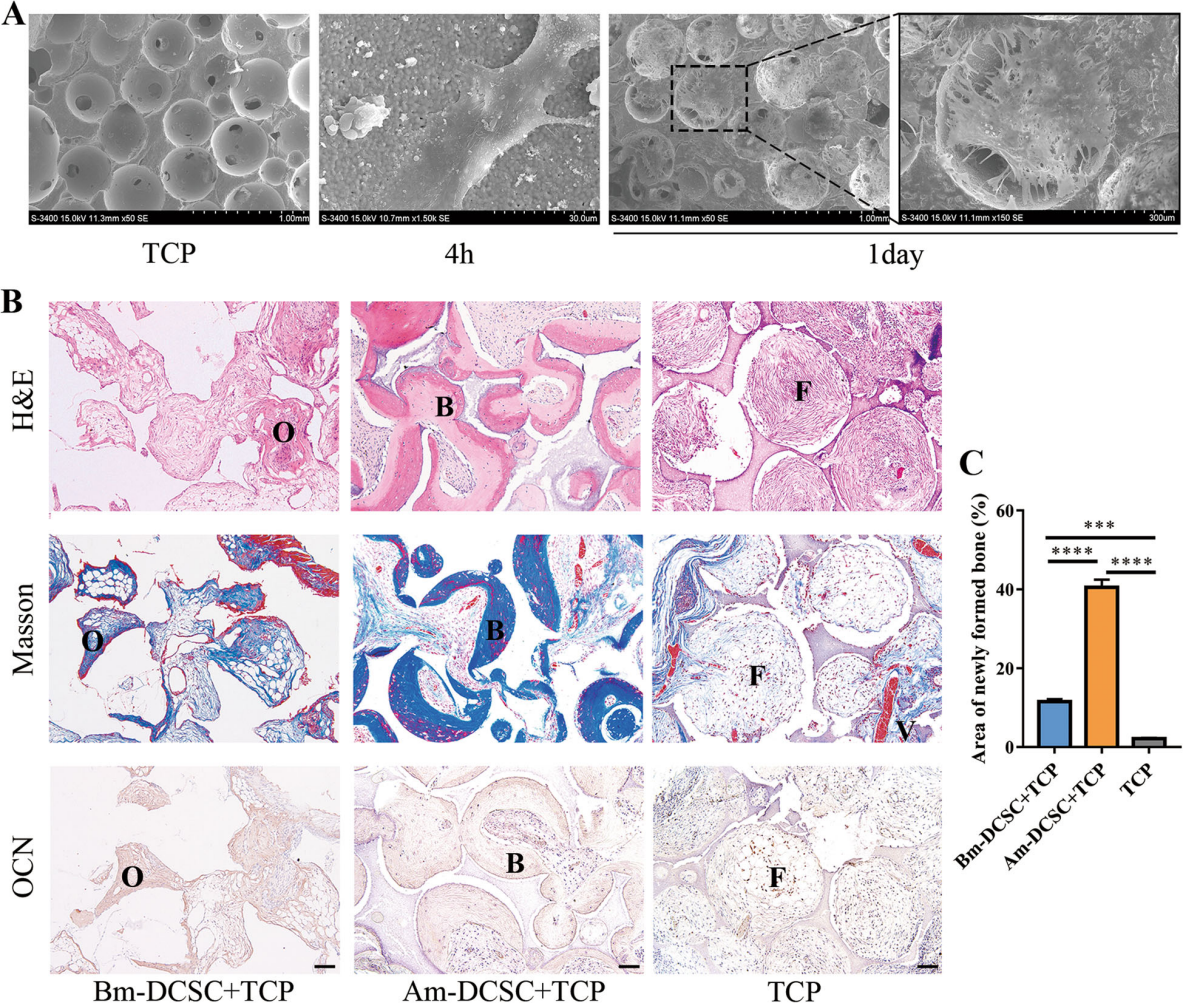
Am-DCSCs showed greater ectopic bone regeneration capacity than that of Bm-DCSCs in vivo. **a** Scanning electron microscopic (SEM) evaluation of the scaffold microstructure and biocompatibility. **b** Histologic analysis of transplants was performed by hematoxylin and eosin staining (H&E), Masson’s trichrome staining, and immunohistochemistry staining for OCN. O: osteoid; B: bone; F: fiber; V: vessel. Scale bars: 100 μm. **c** Quantification of new bone formation area using NIH Image J. ***p < 0.001; ****p < 0.0001

To compare the differences of osteogenic capacity between Am-DCSCs and Bm-DCSCs in vivo, we transplanted them with β-TCP in immunocompromised rats, respectively. After 4 h of incubation, cells attached to β-TCP, and they could spread on the surface 1 day later, which demonstrated that the scaffolds were suitable for the adhesion of Bm-DCSCs and Am-DCSCs (Fig. 6a). After 8 weeks’ implantation, there was a substantial number of the bone tissue in the Am-DCSC group, as collagen bone matrix deposition was illustrated by Masson’s trichrome staining and the mineral deposition was detected by immunohistochemistry (IHC) staining for OCN. Lacunae and osteocytes in it were observed from the H&E staining of the bone tissue, and vessel formation was seen as the evidence of angiogenesis (Fig. 6b). In contrast, only a few osteoid-like tissues were detected in the group of Bm-DCSCs. The results above highlighted the greater osteogenic capacity of Am-DCSCs than that of Bm-DCSCs in vivo.

### Am-DCSCs showed greater bone defect repairability than that of Bm-DCSCs

**Fig. 7:**
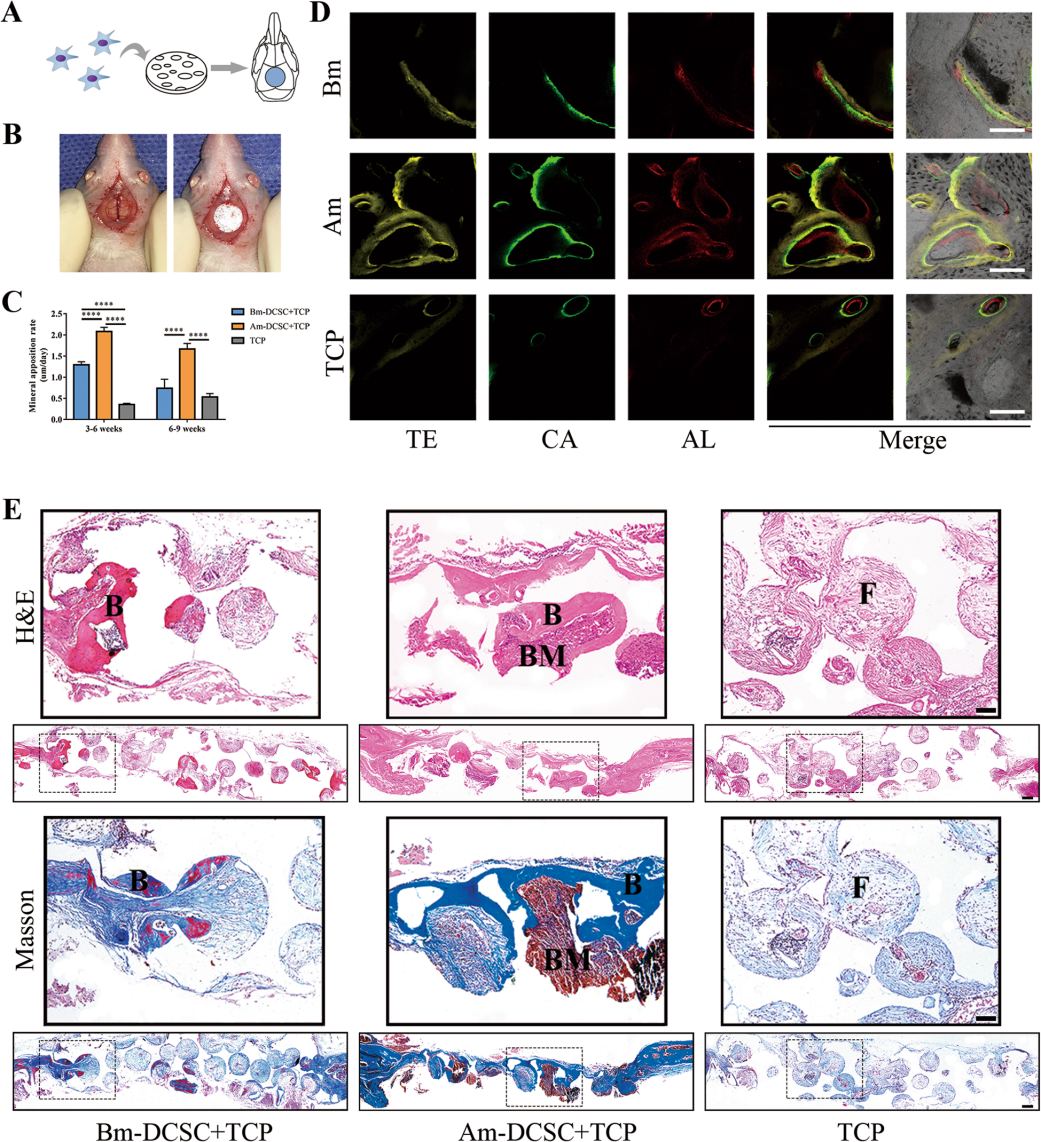
Am-DCSCs showed a greater bone defect repairability than that of Bm-DCSCs. **a** Schematic diagram of animal surgery. **b** Surgery process. **c** Mineral apposition rate (μm/day) was measured by NIH Image J. ****p < 0.0001. **d** Sequential fluorescent labeling images of newly formed bone labeled with hydrochloride tetracycline (yellow), calcein (green), and Alizarin Red S (red). Scale bars: 100 μm. **e** Decalcified sections were stained with H&E and Masson. B bone, BM bone marrow, F fiber. Figures upper are shown at higher magnification. Scale bars: 100 μm and 200 μm

We employed the cranial bone defect model in nude mice to further investigate the bone defect repair capacity of Am-DCSCs and Bm-DCSCs in vivo (Fig. 7a, b). The results visualized in sequential fluorescent labeling showed more and faster new bone formation in the Am-DCSC group than that in the Bm-DCSC group (Fig. 7c, d). Besides, from the H&E and Masson’s trichrome staining of the sections (Fig. 7e), we observed only a few newly formed bone islands embedded in the collagen fiber in the Bm-DCSC group, while newly formed bone tissue was found to directly integrate with and bridge the scaffolds with the negative bone in the group of Am-DCSCs, and the bone-marrow-like structure was observed in new bone formation areas as well. Thus, Am-DCSCs performed better than Bm-DCSCs in bone defect repairment.

## Discussion

In this study, we isolated two types of MSCs from fibrous capsules of DC before and after marsupialization. We identified that both of them showed fibroblast-like appearance, MSC surface markers, and multi-differentiation potential. Compared with Bm-DCSCs, Am-DCSCs showed better proliferation, self-renewal, ectopic osteogenesis, and bone defect repair capacity in vivo. The results above highlighted that there were MSCs residing in capsules of DC, and marsupialization improved their osteogenic capacity both in vitro and in vivo, which may provide a novel insight into the mechanism of bone regeneration after marsupialization.

DC originates from the overexpression of dental lamina in the embryonic period, which become remnants present in the dental arch after birth [27, 28]. When under some unknown initiating factors, odontogenic epithelium enters into an active proliferative phase, then dead cells desquamating into the cyst, plus serum’s diffusion through cyst wall and intra-cystic secretion, resulting in accumulation of fluid and bone-resorbing factors [29]. Marsupialization is a surgical technique by which a window is produced in the wall of the cyst to relieve the intra-cystic pressure and so enable the cavity to decrease slowly in size, then complete enucleation will be performed as a second-stage procedure to reduce recurrence rate [30]. To date, a large amount of literature has proved that marsupialization is an effective method for treating large odontogenic cysts [31–33]. However, this period ranges from 6 to 14 months on average, which seems too long because of the limited osteogenic capacity of the jaw [34].

Fortunately, the stem cell-based bone tissue engineering offers a promising strategy for alveolar bone regeneration [35]. Oral tissues that are rich in stem cells have been thought of as important sources in regenerative medicine [36]. The capsules of DC initially originate from the odontogenic epithelium in the embryonic period, which means it is likely that naive cells hide in them. Bone regenerate after marsupialization with capsules maintaining, suggesting a transition from the osteolytic microenvironment to the osteogenic microenvironment in DC capsules [37]. It would be of major clinical importance if stem cells can be isolated from DC capsules and demonstrated their potential osteogenic capacity in bone defect repair.

Once DC be opened, intra-cystic pressure and fluid containing bone resorption components will be released. Meanwhile, the histological characteristics of cystic linings were confirmed to change after marsupialization, and bone regeneration occurs as follows under the comprehensive effects of various factors. In our study, we collected DC capsules before and after marsupialization, respectively. From the section of Am-DCC, we detected clumps which were demonstrated as immature bone tissue later in the connective tissue layer. In addition, the soft tissue around the clumps was positive for ALP, an early marker of bone formation and produced by osteoblasts, suggesting that the connective tissue of Am-DCC was a microenvironment conducive to bone formation. It is osteoblasts that are responsible for the new bone formation, but where did the osteoblasts in the connective tissue layer of Am-DCC come from?

Although the exact origin of osteoblasts is still under debate, the role of MSCs that play in the osteogenic differentiation has been proved [38]. STRO-1 is a marker for cells in the vicinity of the blood vessels, which has been widely used to identify dental stem cell niches, such as PDLSC, GMSC, and DPSC [12, 39–41]. We also observed STRO-1-positive cells in the connective tissue layer of Am-DCC, which were located around the immature bone tissue. Moreover, cells isolated from Am-DCC (Am-DCSCs) were positive for STRO-1 as well. Given that STRO-1 is an early MSC marker, these preliminary results suggested that the STRO-1 positive cells might be MSCs and could turn into osteoblasts to play a role in the process of new bone formation under certain conditions.

MSCs are a heterogeneous population of cells that show fibroblast-like appearance, plastic-adherent feature, and colony-forming capacity when grown at low densities and multi-differentiation potential [42]. After digestion, Am-DCSCs were adherent to the plastic surface, and they showed a fibroblast-like appearance with elongated, spindle-shaped morphology. Aside from STRO-1, they were positive for mesenchymal stem cell markers like CD44 and CD90, while they were negative for hematopoietic stem cell markers such as CD31, CD34, and CD45 according to FCM results. Actually, we isolated cells from capsules before marsupialization as well. These cells, named as Bm-DCSCs, were also demonstrated as MSCs because of their typical MSC morphological appearance and surface markers.

According to the minimal criteria of MSC proposed by the International Society for Cellular Therapy (ISCT), MSCs must be able to differentiate to osteoblasts, adipocytes, and chondroblasts under standard in vitro conditions [43]. Specifically, both Bm-DCSCs and Am-DCSCs exhibited full tri-lineage potential in vitro, as they were positive for ALP, ARS, Oil Red O, and Alcian blue staining under standard in vitro tissue culture-differentiating conditions. Of note, the osteogenic capacity of Am-DCSCs in vitro was significantly higher than that of Bm-DCSCs at gene, protein, and phenotype expression level. ALP is the specific enzyme secreted by osteoblasts at the early stage of osteoblast differentiation, and calcium nodules stained by ARS is the late osteogenic marker. The relative activity of ALP and ARS showed that Am-DCSCs displayed a greater osteogenic capacity than Bm-DCSCs at the later stage of osteo-differentiation period (day 7 and days 14 and 21), which were consistent with mRNA as well as the osteogenesis-related protein expression of RUNX2 and OCN.

The results of in vivo implantation were analogous to those of in vitro studies. In contrast to the results that only osteoid was observed in the group of Bm-DCSC, the obvious new bone formation could be detected in the Am-DCSC group in the model of subcutaneous ectopic osteogenesis in nude mice. The cranial bone defect model in nude mice was established to further evaluate the potential therapeutic use of Bm-DCSCs and Am-DCSCs. Although Bm-DCSCs could repair bone defect to some extent, Am-DCSCs achieved a better regenerative effect.

Besides, it is known to all that MSCs have a great propensity for ex vivo expansion. Our data revealed that Am-DCSCs had greater proliferation and self-renewal capacity compared with Bm-DCSCs. This is consistent with the observation in orofacial bone marrow stromal cells (BMSCs) around the cysts before and after marsupialization in a previous study [44].

The self-renewal and cell fate decisions of MSCs are sensitive to changes in the extracellular environment. The changed microenvironment after marsupialization, mostly the decreased intra-cystic pressure, is involved in the phenotype changes between Bm-DCSCs and Am-DCSCs. Reports have revealed that mechanical signals from the environment guide basic cellular processes, such as cell survival, proliferation, stem cell lineage commitment, and epithelial to mesenchymal transition (EMT) [45]. EMT is a phenotypic shift in which epithelial cells lose or loosen attachments to their neighbors and assume MSC-like morphology, which leads to cell functional changes [46]. Therefore, we speculate that there are MSCs (Bm-DCSCs) residing in DC capsules, and they are activated and undergo phenotypic changes into Am-DCSCs under the influence of altered microenvironmental factors. Besides, the decreased mechanical pressure induces EMT in DC capsules. For example, epithelial cells, such as lining epithelium cells of DC and oral mucosa epithelial cells, transform into MSCs (Am-DCSCs). This kind of transformation may endow Bm-DCSCs with greater proliferation and osteogenic capabilities. Moreover, the bone resorption environment is broken once DC opened, and osteogenesis dominates in this period. Osteocytes have been demonstrated as both mechanosensory and endocrine cells [47]. Rochefort et al. have reported that osteocytes are able to recruit MSCs via the secretion of OPN to induce new bone formation [48]. It is consistent with the observation by Hu et al. that OPN was upregulated in the keratocystic odontogenic tumor (KCOT) capsule wall after marsupialization [23]. In conclusion, we believe that the activation of MSCs in DC capsules is the consequence of multiple factors, and further research is needed regarding this matter.

Multidisciplinary treatment has attracted extensive attention nowadays because it is conducive to maximize the professional advantages of multiple disciplines in order to reach the best medical effect for patients. The multidisciplinary approach for DC includes marsupialization, orthodontic treatment, and implant placement. That the discovery of MSCs in DC capsules might provide insight to current therapies as follows. Firstly, it provides a scientific basis for the treatment of DC after marsupialization. Secondly, it suggests us to apply cytokine therapy to the treatment of giant DC after marsupialization to accelerate the proliferation and osteogenic differentiation of MSCs, thus finally shortening the healing time of DC, which will pave the way for the next treatment.

## Conclusion

In summary, we have provided evidences that there are MSCs in DC capsules both before and after marsupialization (Bm-DCSCs and Am-DCSCs), confirming that marsupialization improved the proliferation rate and osteogenic capacity of MSCs in DC capsules. Nevertheless, more work needs to be done to explore the underlying mechanisms of MSC phenotype changes in DC capsules and the value of this kind of transformation in the multidisciplinary treatment of DC.

## Data Availability

All data generated or analyzed during this study are included in this article.

## Abbreviations

DC: Dentigerous cyst
MSCs: Mesenchymal stem cells
CCK-8: Cell Counting Kit-8
CFU-F: Fibroblast colony-forming units
EdU: 5-Ethynyl-2-deoxyuridine
ALP: Alkaline phosphatase
ARS: Alizarin Red staining
RT-PCR: Real-time polymerase chain reaction
IF: Immunofluorescence
Am-DCSCs: MSCs isolated from dentigerous cyst capsules after marsupialization
Bm-DCSCs: MSCs isolated from dentigerous cyst capsules before marsupialization
CEJ: Cemento-enamel junction
DPSC: Dental pulp stem cell
PDLSC: Periodontal ligament stem cell
SCAP: Stem cells from apical papilla
Oct-4: Octamer-binding transcription factor 4
K15: Keratin 15
BMP2: Bone morphogenetic proteins-2
BMP4: Bone morphogenetic proteins-4
OPN: Osteoprotegerin
DMEM: Dulbecco’s modified Eagle’s medium
H&E: Hematoxylin and eosin
COL1A1: Collagen type I alpha 1
DAPI: 4′,6-Diamidino-2-phenylindole
PBS: Phosphate-buffered saline
FBS: Fetal bovine serum
EDTA: Ethylenediaminetetraacetic acid
FITC: Fluorescein isothiocyanate
GAPDH: Glyceraldehyde 3-phosphate dehydrogenase
OCN: Osteocalcin
RUNX2: Runt-related transcription factor 2
β-TCP: β-Tricalcium phosphate
TE: Hydrochloride tetracycline
CA: Calcein
AL: Alizarin Red S
Am-DCC: Dentigerous cyst capsule after marsupialization
FCM: Flow cytometry
IHC: Immunohistochemistry
GMSC: Gingiva-derived mesenchymal stem cell
ISCT: International Society for Cellular Therapy
mRNA: Messenger RNA
BMSCs: Bone marrow stromal cells
EMT: Epithelial to mesenchymal transition
KCOT: Keratocystic odontogenic tumor

## References

[1] Meningaud J-P, Oprean N, Pitak-Arnnop P, Bertrand J-C (2006). Odontogenic cysts: a clinical study of 695 cases. J Oral Sci.

[2] Celebi N, Canakci GY, Sakin C, Kurt G, Alkan A (2015). Combined orthodontic and surgical therapy for a deeply impacted third molar related with a dentigerous cyst. J Maxillofac Oral Surg.

[3] Mhaske S (2010). Cysts of the orofacial region.

[4] Aboujaoude S, Ziade M, Aoun G (2020). Five years follow-up of a spontaneous eruption of an impacted mandibular premolar associated with a dentigerous cyst treated by marsupialization. Cureus.

[5] Berti SA, Pompermayer AB, Couto Souza PH, Tanaka OM, Westphalen VPD, Westphalen FH (2010). Spontaneous eruption of a canine after marsupialization of an infected dentigerous cyst. Am J Orthod Dentofac Orthop.

[6] Maltoni I, Maltoni M, Santucci G, Ramina F, Lombardo L, Siciliani G (2019). Marsupialization of a dentigerous cyst followed by orthodontic traction of two retained teeth: a case report. Int Orthod.

[7] Riachi F, Khairallah CM, Ghosn N, Berberi AN (2019). Cyst volume changes measured with a 3D reconstruction after decompression of a mandibular dentigerous cyst with an impacted third molar. Clin Pract.

[8] Lim H-K, Kim J-W, Lee U-L, Kim J-W, Lee H (2017). Risk factor analysis of graft failure with concomitant cyst enucleation of the jaw bone: a retrospective multicenter study. J Oral Maxillo Surg.

[9] Seong JM, Kim B-C, Park J-H, Kwon IK, Mantalaris A, Hwang Y-S (2010). Stem cells in bone tissue engineering. Biomed Mater.

[10] Gronthos S, Mankani M, Brahim J, Robey PG, Shi S (2000). Postnatal human dental pulp stem cells (DPSCs) in vitro and in vivo. Proc Natl Acad Sci U S A.

[11] Morsczeck C, Götz W, Schierholz J, Zeilhofer F, Kühn U, Möhl C (2005). Isolation of precursor cells (PCs) from human dental follicle of wisdom teeth. Matrix Biol.

[12] Seo B-M, Miura M, Gronthos S, Bartold PM, Batouli S, Brahim J (2004). Investigation of multipotent postnatal stem cells from human periodontal ligament. Lancet..

[13] Jussila M, Thesleff I (2012). Signaling networks regulating tooth organogenesis and regeneration, and the specification of dental mesenchymal and epithelial cell lineages. Cold Spring Harb Perspect Biol.

[14] Balic A (2019). Concise review: cellular and molecular mechanisms regulation of tooth initiation. Stem Cells.

[15] Chang JYF, Wang C, Jin C, Yang C, Huang Y, Liu J, et al. Self-renewal and multilineage differentiation of mouse dental epithelial stem cells. Stem Cell Res. 2013;11(3).990–1002. 10.1016/j.scr.2013.06.008 PMC3952636 23906788

[16] Harada H, Mitsuyasu T, Toyono T, Toyoshima K (2002). Epithelial stem cells in teeth. Odontology.

[17] Kurosaka H, Islam MN, Kuremoto K-I, Hayano S, Nakamura M, Kawanabe N (2011). Core binding factor beta functions in the maintenance of stem cells and orchestrates continuous proliferation and differentiation in mouse incisors. Stem Cells.

[18] Alsaegh MA, Altaie AM, Zhu S (2019). Expression of keratin 15 in dentigerous cyst, odontogenic keratocyst and ameloblastoma. Mol Clin Oncol.

[19] Monroy EAC, de Andrade Santos PP, de Sousa Lopes MLD, Mosqueda-Taylor A, Pinto LP, de Souza LB (2018). Oct-4 and CD44 in epithelial stem cells like of benign odontogenic lesions. Histochem Cell Biol.

[20] Padma Priya S, Higuchi A, Abu Fanas S, Pooi Ling M, Kumari Neela V, Sunil PM (2015). Odontogenic epithelial stem cells: hidden sources. Lab Investig.

[21] Marrelli M, Paduano F, Tatullo M (2013). Cells isolated from human periapical cysts express mesenchymal stem cell-like properties. Int J Biol Sci.

[22] Mendes RA, Carvalho JFC, van der Waal I (2010). Characterization and management of the keratocystic odontogenic tumor in relation to its histopathological and biological features. Oral Oncol.

[23] Hu X, Zhao Y, Man Q-W, Li R-F, Liu B, Zhao Y-F (2017). The effects of marsupialization on bone regeneration adjacent to keratocystic odontogenic tumors, and the mechanisms involved. J Oral Sci.

[24] Parfitt AM, Drezner MK, Glorieux FH, Kanis JA, Malluche H, Meunier PJ, et al. Bone histomorphometry: standardization of nomenclature, symbols, and units. . Report of the ASBMR Histomorphometry Nomenclature Committee. J Bone Miner Res 1987;2(6):595–610. 10.1002/jbmr.5650020617 3455637

[25] Schilling T, Mueller M, Minne HW, Ziegler R (1992). Mineral apposition rate in rat cortical bone: physiologic differences in different sites of the same tibia. J Bone Miner Res.

[26] Wronski TJ, Smith JM, Jee WS (1981). Variations in mineral apposition rate of trabecular bone within the beagle skeleton. Calcif Tissue Int.

[27] Hovorakova M, Lesot H, Peterka M, Peterkova R (2018). Early development of the human dentition revisited. J Anat.

[28] Tan B, Tay SY, Shermin L, Teck KC, Yoke PC, Goh C (2013). Malignant transformation of keratocystic odontogenic tumor: two case reports. Am J Otolaryngol.

[29] Suzuki M (1975). A study of biological chemistry on the nature of jaw cysts. On the maintainance of homoeostasis in jaw cyst fluid. J Maxillofac Surg.

[30] Slusarenko da Silva Y, Stoelinga PJW, Naclério-Homem MDG. Recurrence of nonsyndromic odontogenic keratocyst after marsupialization and delayed enucleation vs. enucleation alone: a systematic review and meta-analysis. Oral Maxillofac Surg. 2019;23(1):1–11. 10.1007/s10006-018-0737-3 30498866

[31] Borgonovo AE, Di Lascia S, Grossi G, Maiorana C (2011). Two-stage treatment protocol of keratocystic odontogenic tumour in young patients with Gorlin-Goltz syndrome: marsupialization and later enucleation with peripheral ostectomy. A 5-year-follow-up experience. Int J Pediatr Otorhinolaryngol.

[32] Pogrel MA, Jordan RCK. Marsupialization as a definitive treatment for the odontogenic keratocyst. J Oral Maxillo Surg. 2004;62(6):651–5; discussion 655–6. 10.1016/j.joms.2003.08.029 15170272

[33] Tanaka OM, Meira TM, Batista Rodrigues AC, Willems G, Baggio GL, Couto Souza PH (2019). Marsupialization of a large radicular cyst with extensive maxillary tooth displacement: eight-year follow-up. J Dent Child (Chic).

[34] Zhao Y, Liu B, Han Q-B, Wang S-P, Wang Y-N (2011). Changes in bone density and cyst volume after marsupialization of mandibular odontogenic keratocysts (keratocystic odontogenic tumors). J Oral Maxillo Surg.

[35] Meijer GJ, de Bruijn JD, Koole R, van Blitterswijk CA (2007). Cell-based bone tissue engineering. PLoS Med.

[36] Spagnuolo G, Codispoti B, Marrelli M, Rengo C, Rengo S, Tatullo M. Commitment of oral-derived stem cells in dental and maxillofacial applications. Dent J (Basel). 2018;6(4):72. 10.3390/dj6040072 PMC6313393 30551556

[37] Kubota Y, Yamashiro T, Oka S, Ninomiya T, Ogata S, Shirasuna K (2004). Relation between size of odontogenic jaw cysts and the pressure of fluid within. Br J Oral Maxillofac Surg.

[38] Zhou Q, Yang C, Yang P (2017). The promotional effect of mesenchymal stem cell homing on bone tissue regeneration. Curr Stem Cell Res Ther.

[39] Carnevale G, Pisciotta A, Riccio M, Bertoni L, De Biasi S, Gibellini L (2018). Human dental pulp stem cells expressing STRO-1, c-kit and CD34 markers in peripheral nerve regeneration. J Tissue Eng Regen Med.

[40] Ranga Rao S, Subbarayan R (2019). Passage-dependent expression of STRO-1 in human gingival mesenchymal stem cells. J Cell Biochem.

[41] Mayo V, Sawatari Y, Huang CYC, Garcia-Godoy F (2014). Neural crest-derived dental stem cells--where we are and where we are going. J Dent.

[42] Vemuri MC, Chase LG, Rao MS (2011). Mesenchymal stem cell assays and applications. Methods Mol Biol.

[43] Dominici M, Le Blanc K, Mueller I, Slaper-Cortenbach I, Marini F, Krause D, et al. Minimal criteria for defining multipotent mesenchymal stromal cells. . The International Society for Cellular Therapy position statement Cytotherapy 2006;8(4):315–317. 10.1080/14653240600855905 16923606

[44] Sun Y, Zhang J, Qian N, Sima G, Zhang J, Zhong J (2018). Comparison of the osteogenic differentiation of orofacial bone marrow stromal cells prior to and following marsupialization in patients with odontogenic cyst. Mol Med Rep.

[45] Gjorevski N, Boghaert E, Nelson CM (2012). Regulation of epithelial-mesenchymal transition by transmission of mechanical stress through epithelial tissues. Cancer Microenviron.

[46] Yang J, Antin P, Berx G, Blanpain C, Brabletz T, Bronner M (2020). Guidelines and definitions for research on epithelial-mesenchymal transition. Nat Rev Mol Cell Biol.

[47] Tresguerres FGF, Torres J, López-Quiles J, Hernández G, Vega JA, Tresguerres IF (2020). The osteocyte: a multifunctional cell within the bone. Ann Anat.

[48] Rochefort GY, Pallu S, Benhamou CL (2010). Osteocyte: the unrecognized side of bone tissue. Osteoporos Int.

